# Serum Metabolomic Profiling in Healthy Dogs Supplemented with Increasing Levels of Purified Beta-1,3/1,6-Glucans

**DOI:** 10.3390/ani15091211

**Published:** 2025-04-24

**Authors:** Pedro Henrique Marchi, Leonardo de Andrade Príncipe, Felipe Sesti Trindade, Luana Dias dos Santos, Gabriela Luiza Fagundes Finardi, Eduarda Lorena Fernandes, Thaila Cristina Putarov, Gabriel Henrique Ribeiro, Luiz Alberto Colnago, Júlio Cesar de Carvalho Balieiro, Thiago Henrique Annibale Vendramini

**Affiliations:** 1 Pet Nutrology Research Center (CEPEN Pet), Department of Animal Nutrition and Production, School of Veterinary Medicine and Animal Science, University of Sao Paulo, Pirassununga 13635-900, Brazil; pedro.henrique.marchi@usp.br (P.H.M.); leoprincipe@usp.br (L.d.A.P.); felipesestitrindade@usp.br (F.S.T.); luanads@usp.br (L.D.d.S.); gabriela.finardi@usp.br (G.L.F.F.); eduarda_lorena@usp.br (E.L.F.); balieiro@usp.br (J.C.d.C.B.); 2 Biorigin (Açucareira Quatá S.A.), Lençois Paulistas 18680-900, Brazil; 3 Brazilian Agricultural Research Corporation (Embrapa–CNPDIA), Sao Carlos 13560-970, Brazil; gabrielhenri10@hotmail.com (G.H.R.); luiz.colnago@embrapa.br (L.A.C.)

**Keywords:** canine, metabolome, nutraceuticals, omics sciences, supplementation

## Abstract

Metabolomics has proven to be an effective tool for elucidating mechanisms and assessing the effectiveness of dietary interventions in canine and feline nutrition. In this context, the present study aimed to perform a metabolomic analysis of the serum of dogs supplemented with increasing levels of beta-1,3/1,6-glucans to generate evidence and gain a deeper understanding of the metabolic responses associated with this supplementation. Eight dogs were evenly assigned to two balanced 4 × 4 Latin squares. Four diets were tested, differing only in beta-glucan content (0.0%, 0.07%, 0.14%, and 0.28%), and the dogs were fed according to their individual maintenance energy requirements. Each experimental period lasted 35 days. On day 35, 5 mL of blood was collected via jugular venipuncture to obtain serum for metabolomic analysis. Nuclear magnetic resonance analysis identified 12 key serum metabolites. Hierarchical heat map analysis revealed differences in metabolite intensity between treatments (*p* < 0.05). Additionally, the most relevant metabolic pathways were phenylalanine, tyrosine, and tryptophan metabolism; alanine, aspartate, and glutamate metabolism; and glyoxylate and dicarboxylate metabolism. This study demonstrated that increasing levels of purified beta-1,3/1,6-glucans from *Saccharomyces cerevisiae* modulated key metabolic pathways in dogs, particularly those related to amino acid, lipid and energy metabolisms, and gut microbiota. These findings provide insights into the mechanisms by which beta-glucans influence canine health.

## Simple Summary

Diet plays a crucial role in the health of dogs, and researchers are constantly exploring ways to improve nutrition through dietary supplements. This study investigated how beta-1,3/1,6-glucans, a nutraceutical known for its potential immune and gut health benefits, affect the metabolism of healthy dogs. Eight dogs participated in a controlled experiment using a double Latin square design, ensuring that each animal received all diet treatments in a balanced way. This study lasted a total of 140 days, divided into four experimental periods of 35 days each. The dogs were fed diets with different amounts of beta-glucans (0.0%, 0.07%, 0.14%, and 0.28%), and blood samples were collected at the end of each period to analyze metabolic changes. The results showed that certain levels of beta-glucans (0.07% and 0.14%) had a greater impact on metabolism, particularly in pathways related to amino acids and energy production. These findings help us to better understand how this supplement interacts with a dog’s body and could contribute to the development of more tailored and beneficial diets for pets. Further research is needed to explore the long-term effects and practical applications of beta-glucan supplementation in pet nutrition.

## 1. Introduction

Omics sciences aim to identify, characterize, and quantify the components involved in cellular mechanisms of information and gene expression, from DNA sequences to synthesize proteins and circulating metabolites. This field establishes the connection between an organism’s genotype and its phenotypic manifestation through the application of techniques such as genomics, transcriptomics, proteomics, lipidomics, and metabolomics [1,2,3]. Metabolomics makes it possible to characterize the metabolome of an organ, tissue, or specific sample, which can identify and quantify all of the low-molecular-weight compounds, collectively known as metabolites [4,5].

The metabolome corresponds to the set of metabolites present in a biological system, encompassing well-known molecules such as amino acids, carbohydrates, lipids, and nucleotides, as well as their respective metabolic pathways [6]. In this way, metabolomic analysis provides detailed information on aspects of an animal’s digestive physiology, such as the influence of different ingredients and gut microbiota on metabolism [7]. In this context, metabolomics has proven to be an effective tool for elucidating mechanisms and evaluating the effectiveness of dietary interventions in small animal nutrition [8].

Among the most relevant nutraceuticals for promoting health in dogs are beta-glucans, homopolysaccharides of glucose linked by beta 1→3 glycosidic bonds [9,10]. These bioactive compounds are present in the cellular structure of cereals, fungi, yeast, algae, and bacteria [11,12] and exhibit distinct molecular characteristics and biological functions depending on their source [13]. Among them, beta-glucans extracted from the cell wall of *Saccharomyces cerevisiae* are considered immunostimulant compounds [14]. Their molecular structure is characterized by the predominance of beta 1→3 linkages, with a lower proportion of beta 1→6 linkages and branches [15].

The beta-1,3/1,6-glucans are relevant immunomodulator compounds, with substantial evidence demonstrating their role in enhancing the immune status of dogs and other animals [16]. Due to their structure, they are recognized by specific receptors on myeloid-derived cells, such as dectin-1 [17]. This interaction stimulates innate and adaptive immune responses, activating various immune cells, including neutrophils, B cells, T cells, NK cells, and especially macrophages, while also regulating cytokine production [14,18].

In addition to the immunomodulatory effects, studies conducted by the same research group on the supplementation of beta-1,3/1,6-glucans purified from the cell wall of *Saccharomyces cerevisiae* have shown additional benefits for canine health. These include a reduction in plasma levels of basal glucose, serum cholesterol, and triglycerides in obese dogs, as well as the modulation of the intestinal microbiota in both healthy dogs and those with mild inflammatory bowel disease [19,20,21]. However, the mechanisms by which these compounds exert such metabolic and intestinal effects have yet to be fully elucidated.

In view of this, metabolomic approaches represent a promising strategy for deepening knowledge about the effects of beta-glucans in dogs. Therefore, this study aimed to conduct a metabolomic analysis of the serum of dogs subjected to supplementation with increasing levels of beta-1,3/1,6-glucans to generate evidence and gain a deeper understanding of the metabolic responses associated with this supplementation.

## 2. Materials and Methods

### 2.1. Ethical Statement

This study adhered to the Ethical Principles in Animal Research established by the Ethics Committee on Animal Use of the School of Veterinary Medicine and Animal Science, University of Sao Paulo (CEUA/FMVZ), under protocol number 2866090223.

### 2.2. Animals, Diets, and Experimental Design

The experiment was conducted at the Pet Nutrology Research Center (CEPEN Pet), part of the School of Veterinary Medicine and Animal Science of the University of Sao Paulo, Brazil. Eight dogs from the research facility were used, consisting of four Border Collies and four English Cocker Spaniels, both male and female, aged 3.5 ± 0.5 years, with an ideal body condition score [22]. They were assigned to two contemporary balanced Latin squares with four treatments, according to their breed. Their health status was confirmed through a comprehensive physical examination, nutritional history assessment, complete blood count, and biochemical profile tests, including albumin, glucose, total protein, urea, creatinine, alkaline phosphatase, cholesterol, triglycerides, aspartate aminotransferase (AST), and alanine aminotransferase (ALT).

All dogs used in this study were housed in the same facility, in collective kennels with 3.42 m^2^ of covered area and 7.21 m^2^ of uncovered area, with concrete floors and tiled walls. Dogs were released into grass-covered play areas twice daily to allow for social interaction. Five days prior to blood sampling, the animals were housed individually for fecal sample collection intended for other evaluations and were not released into the play areas during this period. To minimize stress, supervised walks were conducted twice daily in a clean, concrete area. All dogs had ad libitum access to fresh water.

Four nutritionally similar diets were formulated and extruded, differing only in beta-glucan content (0.0%, 0.07%, 0.14%, and 0.28%; Table 1). These levels of beta-glucans were chosen to encompass the doses reported in previous studies [16,19,20,21]. The beta-1,3/1,6-glucan used was a purified extract from the cell wall of *Saccharomyces cerevisiae* with a purity level of at least 60% (MacroGard^®^, Biorigin, Lençois Paulistas, Brazil). The dogs’ maintenance energy requirements were initially calculated using the equation 95 kcal × body weight^0.75^ [23]. This study lasted a total of 140 days, and each experimental period took 35 days, comprising 34 days of diet acclimation and one day for blood sampling. On day 35, 5 mL of blood was collected via jugular venipuncture to obtain serum for metabolomic analysis. Throughout the four periods, blood samples were consistently collected after 12 h of fasting to standardize the metabolic state of all animals.

### 2.3. Metabolomic Analysis

For each sample, an aliquot of 200 µL of serum was diluted in 400 µL of phosphate buffer (D2O-based PBS; 100 mM; pD = 7.4) containing 4.5 mM of sodium 3-(Trimethylsilyl)-1-propanesulfonic acid-d6 (DSS-d6, from Cambridge Isotopes, Leicestershire, UK), used as the nuclear magnetic resonance (NMR) internal standard. Posteriorly, a volume of 600 µL of each sample was transferred to a standard 5 mm NMR tube for NMR measurements. The 1D NMR experiments were performed on a Bruker Avance III 14.1 T spectrometer (Bruker Corporation, Karlsruhe, Baden-Württemberg, Germany) fitted with a 5 mm Broadband Observe probe with ATMA^®^ (Automatic Tunning Matching Adjustment), a z-field gradient, BCU-I variable temperature unit, field gradient generator unit, and a Sample-Xpress™ automatic sample changer.

Standard 1H spectra were acquired at 298.15 K using a NOESY-1D pulse sequence (named noesypr1d in the TopSpin Bruker software version 3.6.1) with water signal suppression by irradiation at 2822.79 Hz (O1). The acquisition parameters were as follows: number scans (ns) of 128, spectral width (sw) of 12,019 Hz (20.0276 ppm), 90° pulse (P1) of 13.53 µs, acquisition time (aq) of 4.52 s, relaxation delay (d1) of 4 s, data points (TD) of 108,544 (106 K), mixing time (d8) of 5 ms, and dummy scans (ds) of 4. The spectra were processed utilizing TopSpin™ 3.6.1 software (Bruker Biospin, Ettlingen, Germany). All the chemical shifts of 1H and 13C were observed in ppm in relation to the sign of DSS in 0.00 ppm as an internal reference, and an exponential enlargement of line of 0.3Hz was applied. The metabolites were assigned based on chemical displacements and multiplied by the signal using the software Chenomx 12.0 [25].

### 2.4. Statistical Analysis

The experimental design used was a contemporary double Latin square with four treatments. The statistical model included the fixed effect of treatment and the random effects of the animal within square, period, and residue. For pre-treatment, simple rectangular buckets of 0.04 ppm were calculated using the Mvova V.15 software (www.mestrelab.com, accessed on 4 February 2024) with the auto-integration mode and scaling to total intensity. The analysis covered regions between 0.2 and 10 ppm, excluding the residual water signal (4.7–5.1 ppm). Metabolome data were uploaded to MetaboAnalyst 5.0 [26], median-normalized, and Pareto-scaled before statistical and bioinformatics analyses (Figures S1 and S2).

The first principal component analysis (PCA, 32 spectra × 235 columns) was performed using the intensity of all obtained metabolites and PERMANOVA validation. Subsequently, key metabolites from the loading analysis of each PC were identified and used for another PCA and hierarchical cluster analysis to compare metabolite intensity between treatments. Additionally, pathway analysis was conducted to identify the most relevant pathways associated with the identified metabolites. Compound names were standardized according to HMDB ID. The pathway analysis parameters included the global test and relative-betweenness centrality, with the Homo sapiens library selected. Metabolic pathways related to the identified metabolites were mapped based on *p*-values (*p* ≤ 0.10) and pathway impacts (impact ≥ 0.001).

## 3. Results

**Figure 1:**
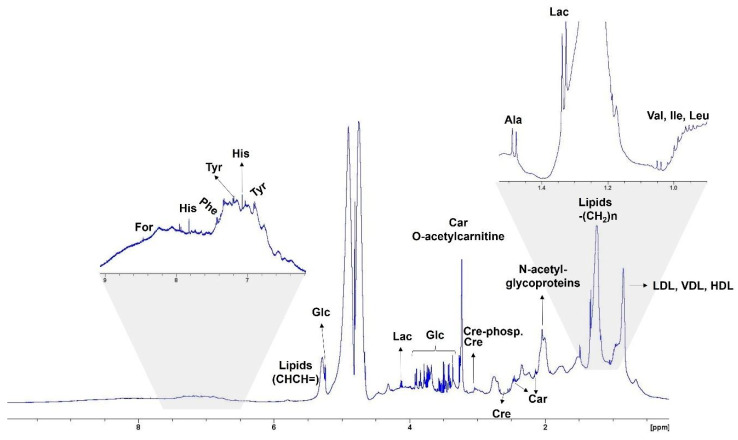
H NMR spectrum of the general pool of dog serum samples. Legend: formate (For); histidine (His); phenylalanine (Phe); tyrosine (Try); glucose (Glc); lactate (Lac); carnitine (Car); creatine (Cre); creatinine phosphate; glutamine (Gln); citrate (Cit); alanine (Ala); isoleucine (Ile); leucine (Leu); threonine (Thr); valine (Val); low-density lipoprotein (LDL); very-low-density lipoprotein (VLD); high-density lipoprotein (HDL).

**Figure 2:**
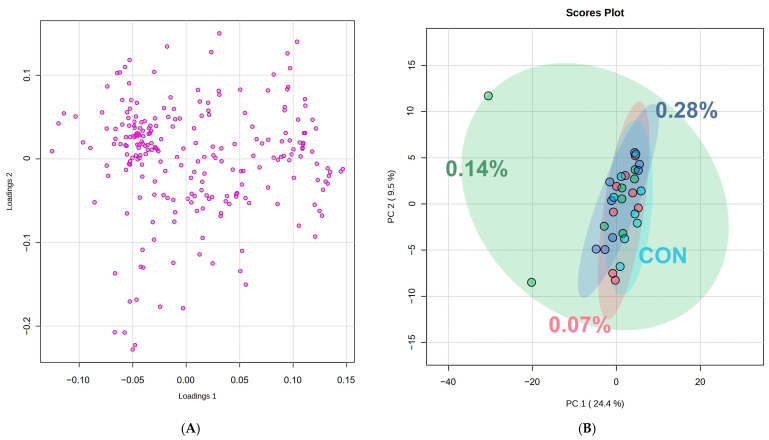
Principal component analysis (PCA) loading plot (**A**) and PCA score plot (**B**) of metabolome distribution between treatments.

Figure 1 represents the spectrum generated by the pooled samples from all experimental units and demonstrates the regions and peaks of the following main serum metabolites: formate, histidine, phenylalanine, tyrosine, glucose, lactate, carnitine, creatine, creatine phosphate, glutamine, citrate, alanine, isoleucine, leucine, threonine, valine, low-density lipoprotein, very-low-density lipoprotein, and high-density lipoprotein. The PCA (Figure 2A) revealed some overlaps between the treatments, in which PC1 explained 24.4% and PC2 explained 9.5% of the total variance of the dataset. Moreover, the PCA loading plot analysis indicated some possible associations between key metabolites and each PC (Figure 2B; Table 1). The top 15 key metabolites observed in PC1 were related to lipids, while those related to PC2 were identified and listed in Table 2.

**Figure 3:**
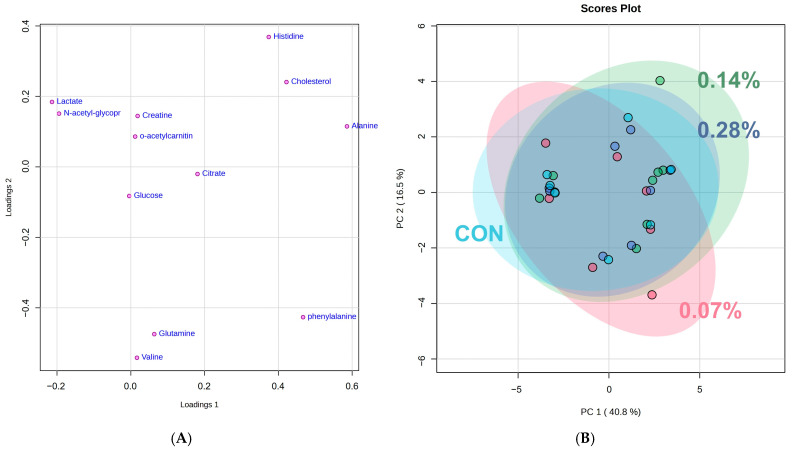
The main analysis components of the main metabolites identified (**A**) and scores plot (**B**).

**Figure 4:**
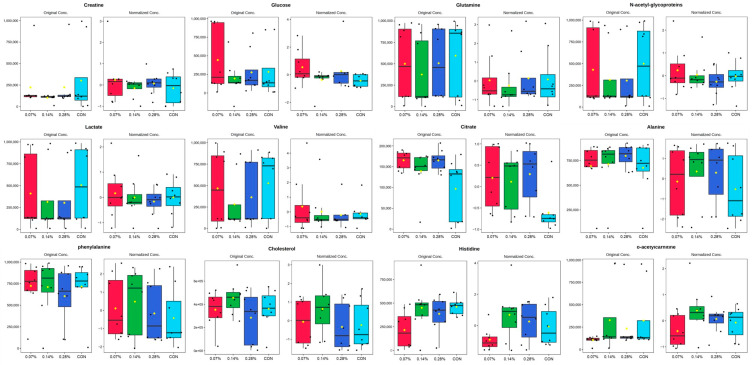
Mean levels of the main metabolites identified before and after normalization.

**Figure 5:**
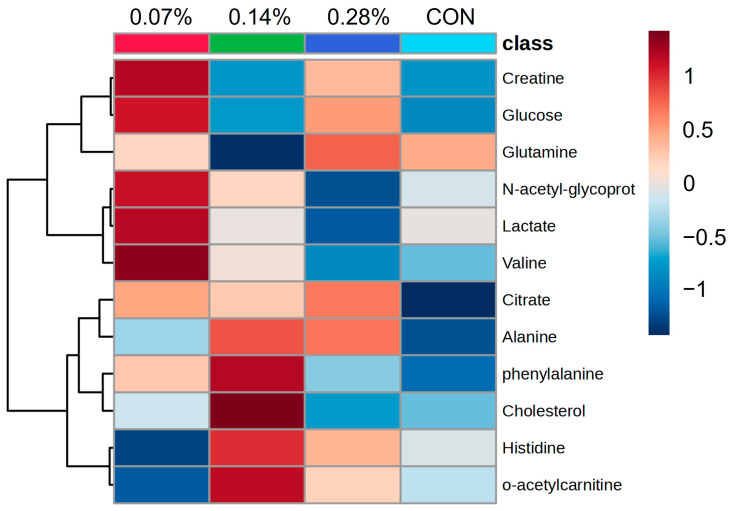
Hierarchical clustering heatmap of identified metabolites. The color scale shown to the right of the heatmap represents the signal intensity of each metabolite. The redder the color, the higher the intensity; the bluer the color, the lower the intensity. The hierarchical clustering does not present statistical significance values.

The complete dataset analysis revealed 15 different lipids associated with PC1 and 12 main metabolites linked to PC2. Based on the 12 identified metabolites, a new PCA was performed (Figure 3A, *p* < 0.05), revealing additional regions of overlap between the treatments. PC1 accounted for 40.8% of the total variance, while PC2 captured 16.5% (Figure 3B, *p* < 0.05). Furthermore, analysis of variance showed no significant differences in metabolite abundance between groups (Figure 4, *p* > 0.05). However, hierarchical heatmap analysis indicated differences in the intensity of the 12 metabolites across the treatments (Figure 5, *p* < 0.05), with the control group displaying greater variability and lower intensity for most compounds compared to the treatment groups.

**Figure 6:**
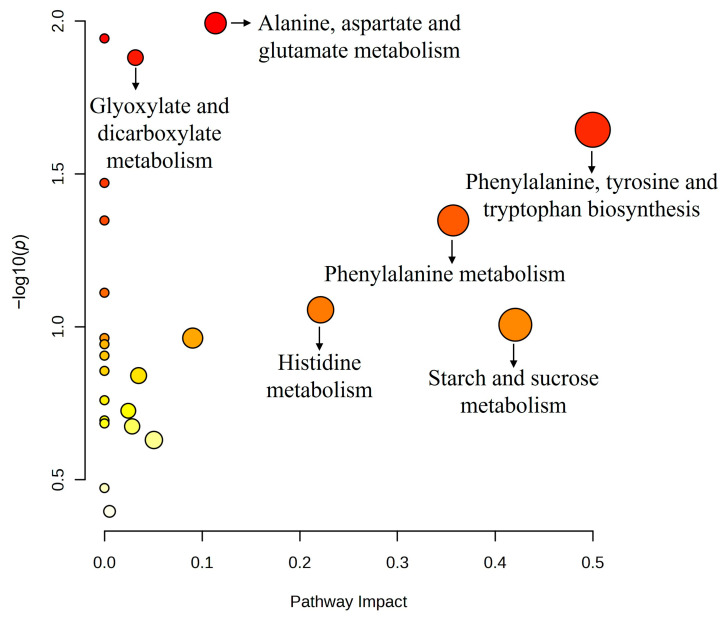
Pathway analysis of the top key compounds identified. In the scatter plot, the *x*-axis indicates the impact on the pathway, whereas the *y*-axis indicates significant changes in the pathway by detected metabolites. Darker nodes represent higher *p*-values from the enrichment analysis, while larger nodes reflect greater impact from the pathway topology analysis. All of the pathways described had *p*-values ≤ 0.10 and pathway impacts ≥ 0.001.

Based on these metabolite intensity differences, an enrichment analysis was conducted to identify the most relevant metabolic pathways (Figure 6, *p* < 0.05). The main pathways are represented by darker nodes (higher *p*-values) and larger sizes (greater impact analysis). All identified pathways had *p*-values ≤ 0.10 and pathway impacts ≥ 0.001 (Table 3, *p* < 0.05). These pathways were associated with protein metabolism—including phenylalanine, histidine, and alanine, aspartate, and glutamate metabolism—as well as phenylalanine, tyrosine, and tryptophan biosynthesis and energy metabolism, which included glyoxylate and dicarboxylate metabolism and starch and sucrose metabolism.

## 4. Discussion

The application of metabolomics to animal nutrition poses challenges in data interpretation due to the large volume of information generated [1]. To address this complexity, different analysis strategies can be employed depending on the study’s aim. The untargeted analysis approach used in this study involves the global profiling of all metabolites present in a sample, without prior selection [27,28]. To organize and facilitate data interpretation, principal component analysis (PCA) is the most commonly used technique for metabolomic data analysis [29]. In this study, the metabolites identified through nuclear magnetic resonance were plotted in loading graphs and evaluated for potential discriminatory patterns between the treatments. In the initial PCA performed using all metabolites from the dataset, a significant overlap was observed. The metabolites with the highest loadings on PC1 were all classified as lipids but could not be individually identified due to technical limitations and the physicochemical characteristics of fats (Han & Gross, 2022) [30]. However, the analysis of PC2 enabled the identification of twelve metabolites with the highest scores, which were subsequently examined in greater detail. The absence of a clear and complete separation in the metabolome profile may be attributed to the subtle inclusion of beta-glucan as the only modified ingredient among the experimental groups. This study was specifically designed to isolate, as much as possible, the effects of beta-glucan inclusion on canine metabolism. With this in mind, it is understandable that a more pronounced separation in the serum metabolome would be unlikely, especially considering that the primary site of beta-glucan action is the intestine. A similar pattern was observed by Ephraim et al. [31], who reported that variations in dietary protein levels did not lead to a clear separation in the metabolite profile.

As a subsequent step, a more detailed analysis was conducted by selecting only the main metabolites. The comparison of mean values before and after the normalization of the twelve most relevant metabolites, along with the overlaps observed in the principal component analysis, indicated minimal differences between the groups. However, hierarchical clustering with a heatmap provided a clearer distinction, highlighting the importance of specific metabolites in differentiating treatments. The heatmap revealed evident differences between the control and beta-glucan groups, where red indicated positive correlations and darker shades of blue represented negative correlations. Overall, all levels of beta-glucan inclusion showed differences in intensity when compared to the control group. Nonetheless, the 0.07% and 0.14% beta-glucan inclusions exhibited the most pronounced differences. This finding corroborates previous studies by our research group, which, although employing different evaluation methods, also concluded that supplementation of approximately 10 mg/kg of beta-glucan promotes beneficial effects in dogs [19,20]. Furthermore, the first results of this experiment indicate that the 0.14% inclusion level (approximately 10.6 mg/kg) led to the best immune response and a favorable fecal microbiota profile [21].

Among the most relevant metabolites, cholesterol and L-acetylcarnitine stood out as being strongly associated with changes in the metabolic profile of dogs in the 0.14% beta-glucan group. Furthermore, the 0.07% and 0.28% groups showed lower signal intensities, characterized by an inverted U-shaped curve. This suggests that the optimal range for beta-glucan inclusion and metabolism lies around 0.14% (or 10 mg/kg), particularly in relation to lipid metabolism. Cholesterol is an essential component of cell membrane structure and lipid metabolism. L-acetylcarnitine plays a crucial role in beta-oxidation, facilitating the transport of acetyl-CoA across mitochondrial membranes for fatty acid oxidation [32]. The interaction between these metabolites suggests a potential influence on lipid metabolism regulation and the physiological response of dogs in the 0.14% beta-glucan group [33,34]. In view of this, studies have shown that beta-glucan inclusion can reduce basal and mean plasma concentrations of glucose, basal insulin, triglycerides, and cholesterol in obese dogs to levels similar to those of healthy dogs [10,19,35]. Incorporating beta-glucans into the diet may be a promising strategy to mitigate dyslipidemia, a condition often associated with hyperinsulinemia and obesity [36].

In addition, this study provided insights into alanine, aspartate, and glutamate metabolism (most significant *p*-value and high pathway impact), highlighted by the production of alanine via the transamination of pyruvate in the presence of glutamate [37]. Alanine plays a crucial role in metabolic regulation, protein synthesis, and ATP supply and can be synthesized from other amino acids, such as valine [38]. In the present study, the heatmap revealed a positive correlation between valine and the 0.07% beta-glucan group. Moreover, these metabolites play a central role in nitrogen metabolism, as compounds such as aspartate, glutamate, and glutamine are synthesized from ammonia during the cycle. Another relevant aspect is the utilization of carbon atoms under extreme conditions for glucose production [37]. Glutamine is involved in virtually all metabolic pathways of proliferating cells [39]. In the present study, glutamine concentration was significantly reduced in the 0.14% group, which may be related to a more pronounced use of this amino acid as an energy source by intestinal cells. Glutamine is the only amino acid extracted from the bloodstream for this purpose and, when metabolized in the intestine, produces alanine [37], which aligns with the observed behavior of both metabolites in the 0.14% group.

In this study, the biosynthesis of phenylalanine, tyrosine, and tryptophan exhibited the highest pathway impact value. These aromatic amino acids are essential for dogs, except for tyrosine, which is considered semi-essential, as it can be synthesized endogenously from phenylalanine when available [40,41]. Their biosynthesis occurs exclusively in microorganisms and plants via the shikimate pathway [42]. Given that beta-glucans can modulate gut microbiota composition [21], it is likely that these changes enhanced the microbial production of aromatic amino acids, which were subsequently absorbed and became metabolically available in the bloodstream of the dogs. This is a significant finding considering the crucial role that these essential amino acids play in canine health. Both phenylalanine and tyrosine are extensively studied for their neurological functions, serving as precursors to neurotransmitters such as norepinephrine, epinephrine, and dopamine [43]. Highlighting its beneficial effects on brain function, Kano et al. [44] observed that oral tyrosine supplementation positively influenced dogs’ cognitive performance, particularly in tasks related to learning and attention. Beyond its neurological role, tyrosine also contributes to various physiological processes, including coat quality and pigmentation [45,46]. A deficiency of tyrosine, or even its precursor phenylalanine, can lead to coat fading, as metabolites such as eumelanin and pheomelanin, the primary melanin pigments, are synthesized through its metabolic pathway [47]. Additionally, L-phenylalanine, a bioactive form of phenylalanine, has been shown to support intestinal barrier function and act as an inhibitor of epithelial alkaline phosphatase, reducing endotoxemia and inflammation [48,49].

Tryptophan metabolites perform various functions in the body, especially in the central nervous system, where they act as precursors of serotonin and melatonin [50,51,52]. However, the percentage of tryptophan converted into serotonin is very low, whereas approximately 90% of this amino acid is metabolized through the kynurenine pathway in mammals [51,53]. Kynurenine is closely linked to immune and inflammatory responses [52]. During inflammatory conditions, such as colitis, metabolites from this pathway can modulate the immune response by activating the aryl hydrocarbon receptor (AhR). The AhR plays a key role in mediating almost all toxicological effects of aromatic hydrocarbons [54]. Additionally, indoles produced through this pathway have been shown to reduce TNF-alpha activity while increasing the expression of the anti-inflammatory cytokine IL-10 [55]. A study on oat-derived beta-glucans suggests that beta-glucan consumption may alter tryptophan metabolism and could be responsible for reducing the incidence of colorectal cancer in rats [56]. In dogs, kynurenic acid inhibits intestinal motility and may exert an anti-inflammatory effect by inhibiting xanthine oxidase [57]. Additionally, Guard et al. [58] identified that dogs with acute diarrhea exhibited a reduction in urinary tryptophan metabolites. In this sense, an increase in intestinal tryptophan biosynthesis may represent an additional mechanism through which beta-glucans modulate immune activity. In contrast, supplementation with oat-derived beta-1,3/1,4-glucans was shown to reduce plasma kynurenine concentrations in dairy calves [59]. Although this represents a divergent finding, it reinforces the complexity of the microbiome–immune-system interaction. Emerging evidence suggests that tryptophan and kynurenine are key intermediates in these pathways, which have been increasingly recognized for their roles in modulating immune and inflammatory responses.

Additionally, beta-glucan consumption appears to have influenced glyoxylate metabolism. Although its pathway impact value was below 0.1, increased activity in this pathway may also be interconnected with the gut microbiota. This cycle involves the utilization of acetate for glucose production and is generally absent in dog metabolism, occurring only in plants and microorganisms [60]. In the study published by Marchi et al. [21], no differences were observed in fecal short-chain fatty acid levels after the consumption of beta-glucans at any concentration. However, there was a significant increase in Erysipelotrichaceae, Ruminococcaceae, Faecalibacterium, and *Prevotella*, which are known producers of short-chain fatty acids [61]. Furthermore, in a study conducted with older hens that evaluated the effects of beta-1,3/1,6-glucan supplementation on the gut microbiome and metabolome, the modulation of several beneficial bacterial families, as well as positive modulation in glyoxylate metabolism, were also observed [62]. Nevertheless, due to limited supporting evidence, it is not possible to establish concrete hypotheses regarding this finding. The application of metabolomics in animal nutrition provides a comprehensive understanding of the effects of specific ingredients and enables the identification of risk factors for various diseases [63,64]. By offering a broader perspective on metabolism, this approach supports the discovery of biomarkers [65]. To better explore the therapeutic potential of the hypotheses discussed in this paper, further studies involving animals with conditions such as obesity, diabetes, or dyslipidemia are needed. Additionally, integrating metabolomic analysis with the assessment of other parameters will allow for a deeper correlation of metabolic alterations, enhancing our understanding of the underlying mechanisms of these diseases and how beta-1,3/1,6-glucans interact with them.

## 5. Conclusions

This study highlighted metabolic changes in dogs that consumed increasing levels of purified beta-1,3/1,6-glucans from the cell wall of *Saccharomyces cerevisiae*. The main modulated pathways included the metabolism of alanine, aspartate, and glutamate; glyoxylate and dicarboxylate; and phenylalanine, tyrosine, and tryptophan. These alterations in lipid and amino acid metabolism, as well as their relationship with gut microbiota, may contribute to a better understanding of the mechanisms of action of beta-glucans on canine health. Moreover, the results indicate a better metabolic response following the consumption of the 0.14% diet (approximately 10 mg/kg), which is consistent with previous findings in the literature.

## Figures and Tables

**Table 1:** Ingredients and chemical composition of the four experimental foods used in this study.

Item	Diets			
	0.0%	0.07%	0.14%	0.28%
Ingredients (%)				
Corn grain	33.26	33.19	33.12	32.98
Common viscera meal	26.38	26.38	26.38	26.38
Broken rice	15.00	15.00	15.00	15.00
Corn gluten	7.99	7.99	7.99	7.99
Beet pulp	4.00	4.00	4.00	4.00
Fish oil	0.82	0.82	0.82	0.82
Potassium chloride	0.42	0.42	0.42	0.42
Mineral and vitamin premix ^1^	0.50	0.50	0.50	0.50
Common salt	0.30	0.30	0.30	0.30
Choline	0.17	0.17	0.17	0.17
Whole egg powder	0.15	0.15	0.15	0.15
Antifungal	0.10	0.10	0.10	0.10
Antioxidant	0.07	0.07	0.07	0.07
Methionine	0.03	0.03	0.03	0.03
Poultry viscera fat	6.81	6.81	6.81	6.81
Swine fat	4.00	4.00	4.00	4.00
Purified beta-1,3/1,6-glucan ^2^	0.00	0.07	0.14	0.28
Total	100	100	100	100
Chemical composition				
Dry matter (%)	93.11	94.31	94.00	93.13
	Chemical composition in dry matter (%)			
Organic matter	92.13	92.07	92.04	91.93
Crude protein	25.25	25.07	27.81	28.24
Fat	17.69	17.82	17.71	17.42
Ash	7.87	7.93	7.96	8.07
Crude fiber	10.17	9.92	10.15	8.28
Nitrogen-free extract ^3^	39.02	39.26	36.37	37.99
Calcium	2.09	2.07	2.13	2.06
Phosphorus	1.19	1.17	1.18	1.17
Metabolizable energy (kcal/g) ^4^	4.10	4.15	4.14	4.09

A total of 0.0% = dog extruded dry food without beta-glucan inclusion; 0.07% = dog extruded dry food with 0.07% beta-glucan inclusion; 0.14% = dog extruded dry food with 0.14% beta-glucan inclusion; 0.28% = dog extruded dry food with 0.28% beta-glucan inclusion; ^1^ Nutrient enrichment per kg: iron 131.57 mg, copper 16.56 mg, manganese 25.26 mg, zinc 135.83, iodine 1.4 mg, selenium 0.36 mg, vitamin A 18,500 IU, vitamin E 134.56 mg, vitamin C 25.16 mg, vitamin D3 1295 IU, vitamin K, 2.66 mg, thiamine 12.26 mg, riboflavin 19.04 mg, pantothenic acid 32.3 mg, niacin 66.7 mg, pyridoxine 11.92 mg, folic acid 2.16 mg, biotin 0.44 mg, and cobalamin 0.81 mg; ^2^ Commercial purified beta-1,3/1,6-glucan with a minimum purity of 60% (MacroGard^®^, Biorigin, Lençois Paulista, Sao Paulo, Brazil); ^3^ Nitrogen-free extract was calculated by the difference in the known macronutrient content; ^4^ Metabolizable energy was estimated according to NRC [24].

**Table 2:** The top 15 key metabolites observed in PC1 were related to lipids, while those related to PC2 are identified and listed here.

PC1			PC2		
Spectral ppm Range	Compound	Score	Spectral ppm Range	Compound	Score
5.92–5.96	Lipids	0.121	0.24–0.28	Lipids	0.150
5.96–6.00	Lipids	0.118	0.52–0.56	Cholesterol	−0.129
6.00–6.04	Lipids	0.118	0.92–0.96	Valine	−0.177
6.04–6.08	Lipids	0.111	1.16–1.20	Lipids	−0.208
6.08–6.12	Lipids	0.111	1.32–1.36	Lactate	−0.223
8.12–8.16	Lipids	−0.125	1.48–1.52	Alanine	0.134
8.16–8.20	Lipids	−0.119	2.00–2.04	N-acetyl glycoproteins	−0.228
8.24–8.28	Lipids	−0.114	2.04–2.08	Glutamine	−0.168
8.36–8.40	Lipids	0.122	2.52–2.56	Citrate	−0.127
8.40–8.44	Lipids	0.132	2.60–2.64	Creatinine	−0.124
9.56–9.60	Lipids	0.106	3.20–3.24	o-acetylcarnitine	−0.137
9.60–9.64	Lipids	0.111	4.48–4.52	Lipids	−0.130
9.64–9.68	Lipids	0.109	5.36–5.40	Glucose	−0.179
9.68–9.72	Lipids	0.109	7.12–7.16	Phenylalanine	−0.125
9.92–9.96	Lipids	0.110	7.92–7.96	Histidine	0.118

**Table 3:** A description of the pathway analysis from the top 12 metabolites, with positive and negative scores in loading analysis to differ between the experimental groups.

Pathway Name	Match Status	Raw *p*	−log(*p*)	Impact
Alanine, aspartate, and glutamate metabolism	2/28	0.010	1.993	0.114
Glyoxylate and dicarboxylate metabolism	2/32	0.013	1.880	0.032
Phenylalanine, tyrosine, and tryptophan biosynthesis	1/4	0.023	1.644	0.500
Phenylalanine metabolism	1/8	0.045	1.348	0.357
Histidine metabolism	1/16	0.088	1.055	0.221
Starch and sucrose metabolism	1/18	0.099	1.007	0.421

## Data Availability

The data presented in this study are available upon request from the corresponding author.

## Supplementary Materials

The following supporting information can be downloaded at: https://www.mdpi.com/article/10.3390/ani15091211/s1, Figure S1: Normalization by median and Pareto scaling; Figure S2: Percentage of residual variance retained from the general principal component analysis based on the number of principal components.
