# Supplementary material for: Serum Metabolomic Profiling in Healthy Dogs Supplemented with Increasing Levels of Purified Beta-1,3/1,6-Glucans
Source: Animals (Basel). 2025 Apr 24;15(9):1211. doi: 10.3390/ani15091211 (PMC12071151; doi:10.3390/ani15091211)
Supplement: Supplementary file 1 [file animals-15-01211-s001.zip › animals-3540874-supplementary.pdf]

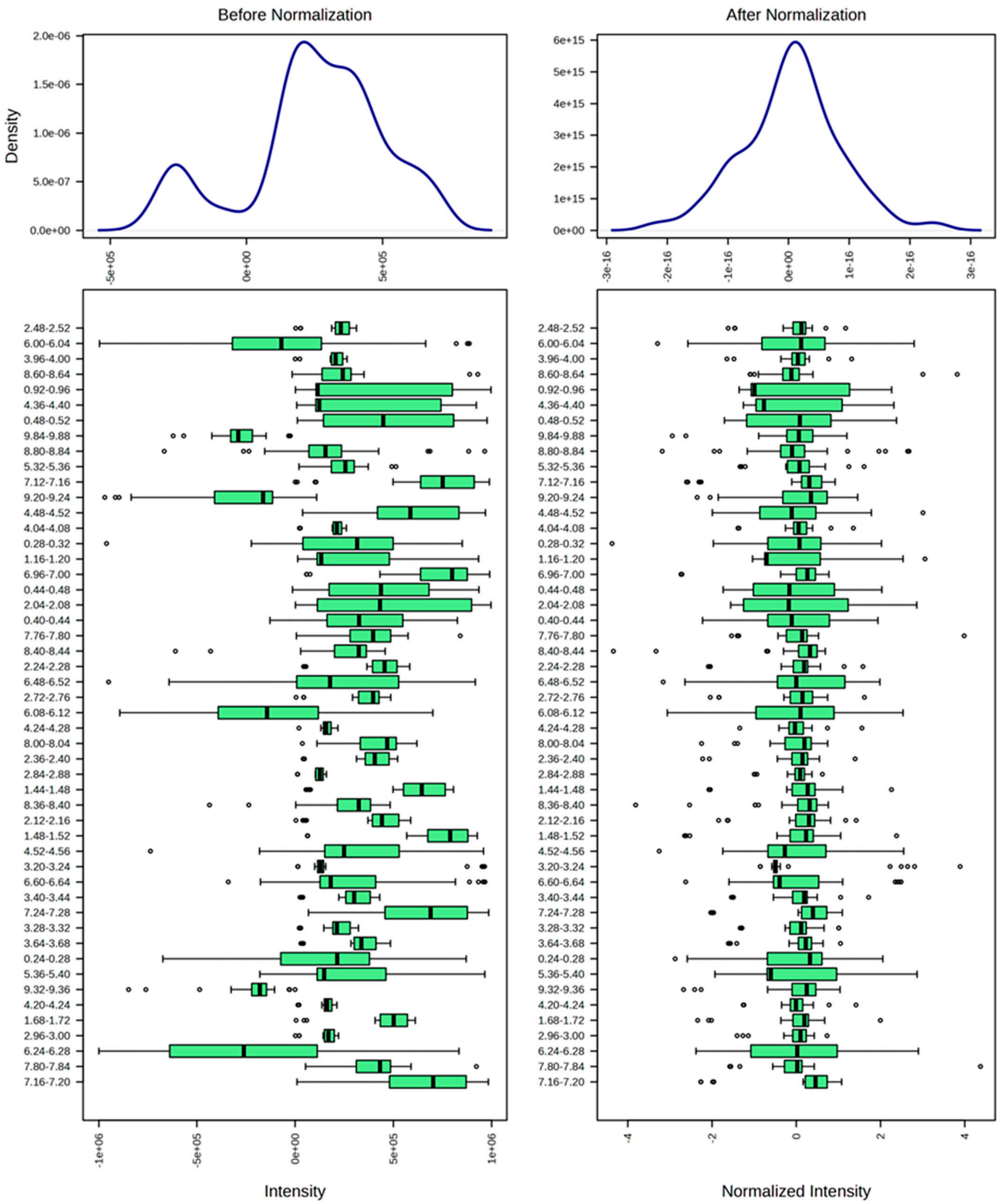

Figure S1. Normalization by median and Pareto scaling.

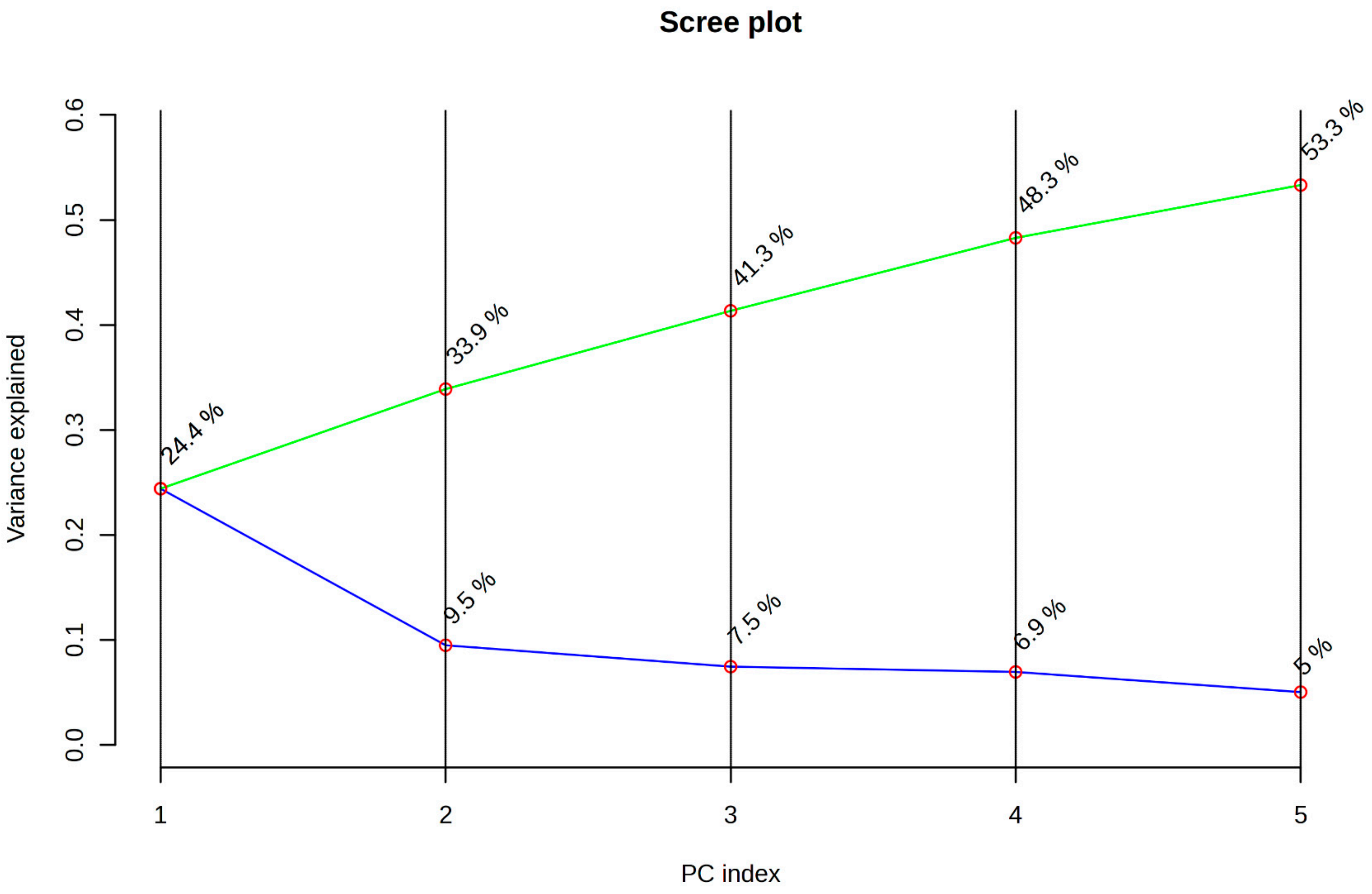

Figure S2. Percentage of residual variance retained from the general principal component analysis based on the number of principal components.
